# The infants’ gut microbiome: setting the stage for the early onset of obesity

**DOI:** 10.3389/fmicb.2024.1371292

**Published:** 2024-07-16

**Authors:** Yvonne Vallès, Muhammad Arshad, Mamoun Abdalbaqi, Claire K. Inman, Amar Ahmad, Nizar Drou, Kristin C. Gunsalus, Raghib Ali, Muna Tahlak, Abdishakur Abdulle

**Affiliations:** ^1^Public Health Research Center, New York University Abu Dhabi, Abu Dhabi, United Arab Emirates; ^2^Core Bioinformatics, New York University Abu Dhabi, Abu Dhabi, United Arab Emirates; ^3^Center for Genomics and Systems Biology, New York University Abu Dhabi, Abu Dhabi, United Arab Emirates; ^4^Department of Biology and Center for Genomics and Systems Biology, New York University, New York, NY, United States; ^5^Latifa Women and Children Hospital, Dubai, United Arab Emirates

**Keywords:** microbiome, obesity, infant, maternal, offspring, early disorder markers

## Abstract

In the past three decades, dietary and lifestyle changes worldwide have resulted in a global increase in the prevalence of obesity in both adults and children. Known to be highly influenced by genetic, environmental and lifestyle factors, obesity is characterized by a low-grade chronic inflammation that contributes to the development of other metabolic diseases such as diabetes and cardiovascular disease. Recently, the gut microbiome has been added as a cause/contributor to the development of obesity. As differences in the microbiome between obese and normoweight individuals have been observed, we set out to determine whether infants harbor an obesogenic microbiome early on and whether the pre-pregnancy status of the mother (obese or normoweight) is correlated to their infant’s microbiome composition. Using shotgun sequencing, we analyzed stool samples throughout the first year of life from infants born to obese (*n* = 23 participants, *m* = 104 samples) and normoweight (*n* = 23 participants, *m* = 99 samples) mothers. We found that the infants’ microbiome diversity at taxonomic and functional levels was significantly influenced by time (ANOVA *p* < 0.001) but not by the mother’s pre-pregnancy status. Overall, no deterministic succession of taxa or functions was observed. However, infants born to obese mothers were found to have a significantly higher Bacillota/Bacteroidota ratio (*p* = 0.02) at six months, were significantly depleted from six months old of the well-established obesity biomarkers *Akkermansia municiphila* and *Faecalibacterium prausnitzii* (*p* < 0.01), and were at one week old, significantly enriched in pathways such as the UDP-N-acetyl-D-glucosamine biosynthesis II (*p* = 0.02) involved in leptin production, suggesting perhaps that there may exist some underlying mechanisms that dictate the development of an obesogenic microbiota early on.

## Introduction

Obesity is a disorder of the modern age whose prevalence has relentlessly and globally increased in the past three decades, with no discrimination to gender, age, or ethnicity (Rubenstein, 2005; WHO, 2021). Obesity is characterized by a low-grade systemic inflammatory state that has been consistently associated in a “dose–response” relationship with diabetes, cardiovascular diseases, some cancers, and respiratory disorders to name a few (Rubenstein, 2005). The global increase in the prevalence of children’s obesity is particularly unsettling, as overweight/obese children are likely to remain overweight/obese as adults and are therefore more prone to develop non-communicable diseases such as diabetes and cardiovascular disorders at a younger age (Rubenstein, 2005; WHO, 2021).

A complex disorder, obesity’s etiology is known to be influenced by genetic, environmental and lifestyle factors (Rubenstein, 2005). Interestingly, the microbiome of the gastrointestinal tract (GIT), a subject of extensive research in the past two and a half decades, has recently been identified as another factor in the etiology of obesity (Kvit and Kharchenko, 2017; Zhang and Dang, 2022). Although there is still controversy regarding universally recognized microbiome differences between obese and normoweight individuals, as discrepant results have been reported (Andoh et al., 2016; Stanislawski et al., 2019; Pinart et al., 2021), many studies have observed in normoweight adults (1) a higher diversity of the GIT microbial community, (2) a lower Bacillota/Bacteroidota ratio, and (3) a higher relative abundance of the genera *Akkermansia* and *Faecalibacterium* and their respective species *Akkermansia municiphila* and *Faecalibacterium prausnitzii* compared to obese adults (Ley et al., 2006; Tap et al., 2009; Turnbaugh and Gordon, 2009; Knights et al., 2011; Payne et al., 2011; Bervoets et al., 2013; Le Chatelier et al., 2013; Ridaura et al., 2013; Patterson et al., 2016; Abenavoli et al., 2019; Maioli et al., 2021). Interestingly, similar differences have also been observed between the GIT microbiomes of normoweight and obese pregnant women (Santacruz et al., 2010; Zacarias et al., 2018; Yang et al., 2020).

The Developmental Origins of Health and Disease (DOHaD) hypothesis states that developing in an unfavorable “*in utero*” environment negatively influences long-term health, thus increasing the risk of developing metabolic disorders such as obesity, diabetes, and cardiovascular disease later on in life (Charles et al., 2016). Considering this, together with the recently recognized role of the GIT microbiome as an etiological factor in the development of obesity and the fact that GIT microbial differences are observed between normoweight and obese pregnant women, we explored whether the infant’s GIT microbiome displays obesogenic characteristics in the early stages of development and whether significant associations between the pre-pregnancy status of the mother (normoweight or obese) and the infant’s GIT microbiome can be identified throughout the first year of life of the infant.

Finally, since modulation of the gut microbiota has been shown to be a potentially powerful therapeutic approach to treat obesity (Le Chatelier et al., 2013; Ridaura et al., 2013; Patterson et al., 2016), understanding whether or not there are significant GIT compositional differences between infants born to pre-pregnancy obese mothers (Ob offspring) and infants born to pre-pregnancy normoweight mothers (Nw offspring) from birth and throughout their first year of life could prove to be useful in the development of early GIT modulation interventions that in turn may help in preventing the early onset of obesity.

## Materials and methods

### Ethics statement

This study was approved by the NYU Abu Dhabi and the Dubai Health Authorities (DHA) Ethics Committees. All women participating in the study read information on the requirements to be part of the study and signed consent forms specifically approved for this project by both Ethics Committees.

### Recruitment and sample collection

Participants were recruited at Latifa Hospital in Dubai during a 19-month period between August 2017 and May 2019. Eligibility to participate in the study entailed being a pregnant Emirati women aged 18 and above, with a pre-pregnancy BMI either normal weight (18.5-24.9) (Turnbaugh and Gordon, 2009; Santacruz et al., 2010; Charles et al., 2016; Lu et al., 2017; Uritskiy et al., 2018; Zacarias et al., 2018; Wood et al., 2019; Yang et al., 2020) or obese (>30) with no other comorbidities or health concerns. To be eligible to participate in the study, the infant’s mothers should not have taken any course of antibiotics from 28 weeks of pregnancy until delivery. In addition to the infants ‘stool samples collected by their respective mothers at each time point, a questionnaire was completed initially providing information on the mothers’ medical and family history and lifestyle, on the delivery mode and gender of the infant, and thereafter, at the time of sample collection, on the infants’ diet, antibiotic intake, and specific household habits such as the use of antibacterial soaps or presence of pets in the household.

Stool samples were collected by the participants through the help and training procured by qualified nurses. Parents of participants were given an OMNIgene-GUT OMR-200 all-in-one system (DNA-Genotek) to collect the stool sample and provide a sample stable environment at the exact moment of collection. Samples were then collected from participants, aliquoted into 1 mL cryovial tubes (Nunc/Thermo, cat #374088) and stored in the lab at −80°C until further processing. Stool samples were collected at 5 different timepoints throughout the first year of life of the participant: at one week, two months, four months, six months and twelve months. After sample collection, sample processing, and subsequent sequencing quality control, a total of 203 samples were included in the study from our 46 participants. A minimum of three samples per participant were required to remain in the study. A breakdown on the samples included in the study is shown in Supplementary Table S1.

### DNA extraction, library preparation and sequencing

Samples were thawed, vortexed and an aliquot of 200 μl was used for DNA extractions. Total DNA was extracted using the Zymo Research Quick DNA kit (cat #D6010) following manufacturer’s instructions. Quality of DNA was assessed through gel electrophoresis and yields were determined using the HS dsDNA assay kit for Qubit (Thermo Fisher Scientific -US) following the manufacturer’s specifications. Total DNA extractions were stored at −80°C until further processing. Libraries were prepared from purified genomic DNA using the NEBNext ultra II FS DNA library prep kit (E7809L) and NEBNext multiplex oligos (E7600S and E7780S) for lllumina following the manufacturers’ specifications. Quality of libraries was evaluated using the Bioanalyzer High Sensitivity DNA Analysis (5067-4626 Agilent Technologies). Libraries were sequenced on a NovaSeq 6,000 platform using the NovaSeq 600 S1 Reagent kit (300 cycles) and the NovaSeq XP 2-lane Kit (cat #20021664 v1 Reagents) following the manufacturers’ recommendations (Illumina).

### Metagenomic reads QC

For metagenomic data analysis, the metaWRAP (v1.3.2) pipeline was used (Uritskiy et al., 2018) as it contains independent modules for shotgun metagenomic data analysis. First, raw reads were quality checked and quality trimmed (QC/QT). This involved trimming reads using Trim-galore to remove sequencing adapter content, as well as low quality (Q score) sequences. Reads were aligned to the human genome (hg38) with bmtagger to remove host contamination. Only clean and high-quality (Phred score > 30) metagenomic reads were used for further downstream analysis (Supplementary Table S2).

### Taxonomic profiling and pathway analysis

Each read pair was taxonomically classified using the k-mer based analysis implemented in kraken 2 [v2.1.2; (Wood et al., 2019)] against the standard database (built on 23-05-2023 with the command: kraken2-build –standard –db standard –threads 24). To estimate taxonomic abundances in each sample at different taxonomic levels, the output of the kraken2 analysis was then analyzed using Bracken (Bayesian Re-estimation of Abundance after Classification with KrakEN) v2.8 (Lu et al., 2017).

To identify metabolic pathways, pathway abundance profiling was performed using HUMAnN v3.5 (HMP Unified Metabolic Analysis Network) (Franzosa et al., 2018). The databases used for this analysis included the ChocoPhlAn (v201901_v31) pangenome and UniRef90 protein database (uniref90_201901b_full). Pathway abundance quantification was performed according to the methods described in (Jo et al., 2020).

### Statistical analyses

Alpha-diversity measures of the infants’ GIT microbiome were assessed using a rarefied data set created in qiime 2 (Caporaso et al., 2010), at both taxonomic and functional levels. Previously, the SRS.shiny.app with default parameters (Heidrich et al., 2021) was used to determine the minimum number of sequences to be included in the rarefied datasets. Statistical significance was ascertained by performing an analysis of variance (ANOVA) on a multiple linear regression model adjusting for pre-pregnancy status, timepoint, delivery mode and gender. Pearson’s Chi-squared test with simulated *p*-values was used to assess the statistical significance of the association between categorical variables and the offspring groups classified as normoweight (Nw) and obese (Ob). Due to low expected frequencies in some cells of the contingency table, 2,000 Monte Carlo simulations were used to approximate the distribution of the Chi-squared statistic under the null hypothesis. Linear regression models were carried out to test for statistically significant associations between the infants’ GIT microbiome and variables of interest [Vegan library (v2.5–2)] (Oksanen et al., 2013). Briefly, models were built including relative abundances for each phylum examined as response variables and pre-pregnancy status, timepoint, delivery mode and gender as predictor variables. A sensitivity analysis was performed by fitting a linear mixed effect model with type, gender and delivery mode as fixed effects variables, and timepoint as a random effect variable. The outcomes of interest were the following phyla: Actinomycetota, Bacillota, Bacteroidota, Pseudomonadota, Fusobacteriota, and Verrucomicrobiota. The analysis was repeated for each of these phyla. Bacillota to Bacteroidota ratios were compared between Ob and Nw offspring using Wilcoxon rank sum test. We conducted a permutational multivariate analysis of variance (PERMANOVA) using Bray-Curtis distance matrices to test significant differences of taxonomic and functional profiles between both infant groups. Canonical Correspondence Analyses (CCA) were used to explore associations between the mother’s pre-pregnancy status, timepoint, gender, and delivery mode, and the infants’ GIT microbial spatial composition. Statistical significance was evaluated using an ANOVA test. Subsequently, differential abundances of taxa and functional pathways were analyzed using the DESeq2 software package (Love et al., 2014), a method specifically tailored for count data derived from high-throughput sequencing technologies. All applied statistical tests were two-sided. *p*-values <0.05 were considered as statistically significant. To control the false discovery rate (FDR) associated with multiple hypothesis testing, a Benjamin-Hochberg adjusted *p*-value of less than 0.05 was considered statistically significant. This adjustment was implemented using DESeq2. All analyses were conducted in R version 4.2.0 (Team RC, 2014).

## Results and discussion

As childhood obesity has become a worldwide concern and the gut microbiome has been credited as being part of the cause, we explored the possibility that the mother’s pre-pregnancy status, obese versus normoweight, could be a factor in the early childhood onset of obesity. General trends of taxa and functional diversity were similar in both contexts, with both richness and diversity mostly increasing with time. Microbial community composition varied among time periods but not between pre-pregnancy groups, revealing a conserved and clear directionality from the earliest to the latest timepoint at both taxonomic and functional levels. Yet, differential abundance analysis revealed expected and interesting differences between Ob and Nw offspring such as the depletion of *Akkermansia municiphila* or the enrichment of the UDP-N-acetyl-D-glucosamine biosynthesis II pathway in Ob offspring.

### Demographics of the Emirati infants’ cohort

Our final cohort comprised 46 Emirati participants and a total of 203 samples. All infants included in the study were born at term (37 weeks or more) with 37% being female and 63% being male. The difference in the mode of delivery between the two groups is statistically significant (*p*-value = 0.0065), with a much higher proportion of Ob offspring born via c-section compared to Nw offspring. This suggests that the mode of delivery could be a factor influencing obesity in offspring, aligning with research that suggests c-section might impact the gut microbiota and subsequently the child’s metabolic health. Other factors such as gender, early feeding practices (e.g., all infants were exclusively breastfed up to their first 2 months of life), maternal and gestational diabetes, mother and child antibiotic consumption and pet exposure did not show significant differences between normoweight and obese groups, suggesting that these may not be critical determinants of obesity in this particular sample. Further research could explore other potential factors or larger sample sizes to validate these findings (Table 1). The establishment of this cohort was an important step towards contributing to filling a gap within the microbiome field in the Middle East, since geographical populations as well as cultural and traditional differences play important roles in shaping microbial assemblages. This study is the first of its kind in the Gulf Cooperative Council (GCC) region.

**Table 1:** Participants characteristics according to the mother’s pre-pregnancy BMI status; with frequencies (percentages) and Pearson’s Chi-square test *p*-values.

Demographic traits [*n* (%)]	Nw offspring (*n* = 23)	Ob offspring (*n* = 23)	*p*-value^*^
Gender			1.000
Female (37%)	9 (20)	8 (17)	
Male (63%)	14 (30)	15 (33)	
Delivery mode			0.0065
C-section (39%)	4 (9)	14 (30)	
Vaginal (59%)	18 (39)	9 (20)	
Not provided (2%)	1 (2)	0 (0)	
Feeding (before 2 months)			0.196
Breastfeeding	17 (37)	20 (44)	
Formula	0 (0)	1 (2)	
Mixed	5 (11)	1 (2)	
Not provided	1 (2)	1 (2)	
Feeding (Introduction of solids)			0.534
At 6 months	10 (22)	14 (30)	
After 6 months	11 (24)	9 (20)	
Not provided	2 (4)	0 (0)	
Diabetes (mother)			1.000
Yes (8%)	2 (4)	2 (4)	
No (74%)	15 (33)	19 (41)	
Not provided (17%)	6 (13)	2 (4)	
Pets at some point in the household thru the first 12 months			1.000
Yes (28%)	6 (13)	7 (15)	
No (72%)	17 (37)	16 (35)	

^*^Simulated *p*-value (based on 2,000 replicates).

### Overall GIT microbial community structure

After quality control, the final taxonomic dataset comprised 28 phyla, 58 classes, 130 orders, 311 families, 871 genera and 1823 species of bacteria. Amongst the phyla observed, five of them comprised the greatest majority of the bacterial population (>97.86%), with Actinomycetota having an average relative abundance of 33.88%, Pseudomonadota 22.80%, Bacillota 18.60%, Bacteroidota 24.01% and Verrucomicrobiota 0.57%. Looking at the phyla relative abundance between Ob and Nw offspring revealed that most differences can be observed early on at one week (Supplementary Figure S1). While our findings are consistent with most studies with respect to the five major phyla, we were surprised to observe that Actinomycetota (usually third most abundant) was the most abundant, and that Pseudomonadota (typically first or second most abundant) was the third most abundant (Arumugam et al., 2011; Lozupone et al., 2012). Because microbiome data from Middle Eastern populations is greatly underrepresented, the differences observed could be reflecting ethnicity and genetic factors as well as the influence of geographical and/or cultural components (Yatsunenko et al., 2012). An example of the latter are dates, a staple fruit of the Emirati diet, which are rich in polyphenols and dietary fiber and are known to increase the abundance of *Bifidobacterium* species (Actinomycetota), in the gut microbiota (Eid et al., 2014), which could potentially be translocated from mother to fetus/infant during pregnancy and breastfeeding.

Both time and delivery mode are established drivers of microbial assemblages (Dominguez-Bello et al., 2010; Valles et al., 2014). In our study, we found that the overall GIT microbiota structure and phyla composition were significantly associated with timepoint and delivery mode (PERMANOVA test, *p* < 0.001), while no significant associations were observed with respect to either the mother’s pre-pregnancy status or gender of the infant. Similarly, timepoint and delivery mode, but not other factors, were identified as significant at lower taxonomic levels (class, order, family, genus and species, data not shown), and the same was true for functional potential (PERMANOVA test *p* < 0.001). A linear regression model with Bacillota as an outcome and timepoint, pre-pregnancy status, delivery mode, and gender as predictors showed statistically significant timepoint differences between one week and twelve months (*p*-value = 0.0008) and in delivery mode (*p*-value = 0.014). In a multivariate model where Bacteroidota was the outcome, statistically significant timepoint differences were observed between one week and four months (*p*-value = 0.046) and one week and twelve months (*p*-value = 0.039) as well as in delivery mode (*p*-value = 0.0028). Actinomycetota and Pseudomnadota showed significant associations only with timepoint, with *p*-values of 0.035 and 4.4e-07 respectively). Supplementary Table S3 shows the estimated effect of pre-pregnancy status, gender and delivery mode with 95% confidence interval (95% CI), derived from the fitted linear mixed effect model in the sensitivity analysis. The result of the sensitivity analysis revealed the same associations as those observed in the main statistical analysis, with the exception of the statistically significant effect of type (pre-pregnancy status) on the phylum Verrucomicrobiota. Supplementary Figure S2 shows the estimated intercept with 95% CI for each phylum by timepoint. The twelve months timepoint was statistically significantly higher than the average for Bacillota. However, for Pseudomandata, four months and twelve months timepoints were significantly lower than the average while the one week timepoint was statistically significantly higher than the average. No statistically significant differences were observed for the remainder phyla.

**Figure 1:**
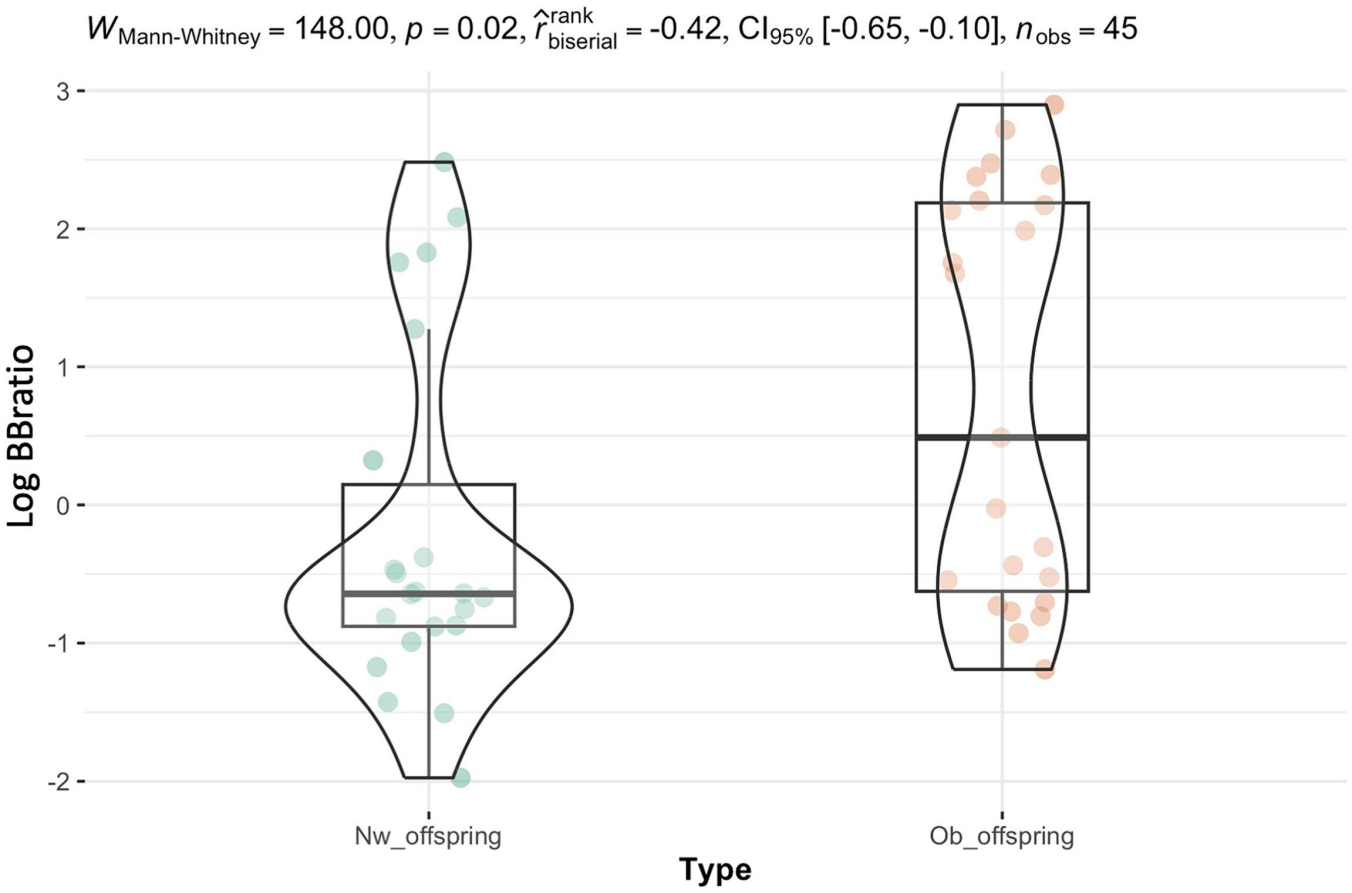
The Bacillota/Bacteroidota ratio was significantly different between Ob and Nw offspring at six months (*p* = 0.02), which concurred with the introduction of solid foods. The y-axis corresponds to the log Bacillota/Bacteroidota ratio.

In addition to the significant effect revealed by the sensitivity analysis of pre-pregnancy status on the phylum Verrucomicrobiota, which supports the knowledge that this phylum is essential to maintain glucose homeostasis in a healthy gut, a notable difference between Ob and Nw infant groups was the significantly higher Bacillota/Bacteroidota ratio observed in Ob offspring at six months, following the introduction to solid foods (*W* = 148.00, *p*-value = 0.02, Figure 1). Interestingly, no significant differences of this ratio were observed at any other timepoints (Supplementary Figure S3).

### Measures of diversity are significantly influenced by time but not the mother’s pre-pregnancy status

**Figure 2:**
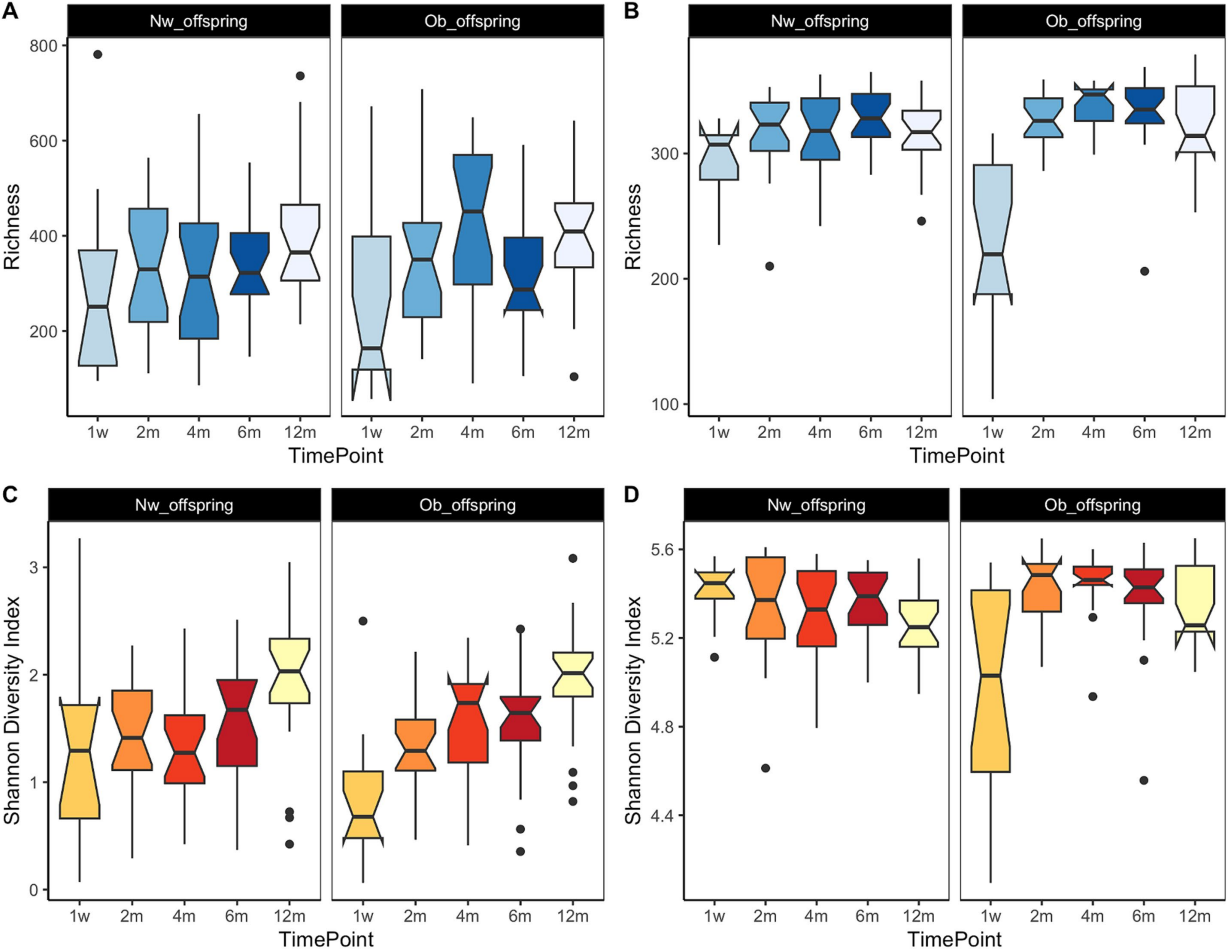
Dynamics of diversity measures throughout the infant’s first year of life. **(A,C)** display Richness and Shannon-Weaver diversity index, respectively, at the taxonomic level while **(B,D)** display the functional potential richness and Shannon-Weaver diversity index, respectively. Time points in the x axis correspond to 1w—one week, 2 m—two months, 4 m—four months, 6 m—six months and 12 m—twelve months. Both richness and the Shannon-Weaver diversity index were significantly associated with time at both taxonomic and functional levels (*p* < 0.001). Similar trends were observed at the functional level, where there was an initial sharp increase of richness and diversity, followed by a plateau from two months to six months and a final slight decrease at twelve months.

**Figure 3:**
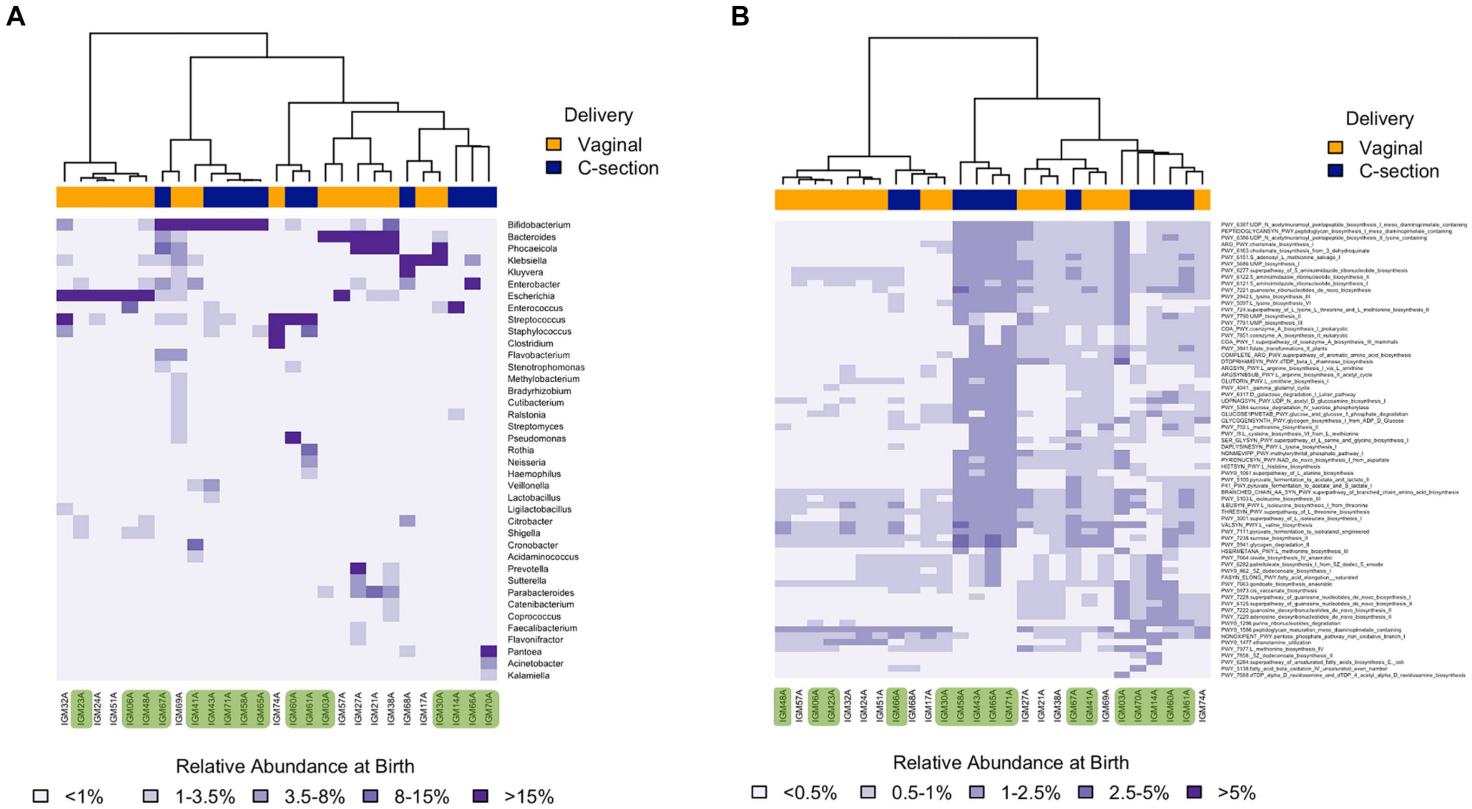
Heatmaps depicting the infants gut microbiome composition based on Bray-Curtis distances at one week at both taxonomic **(A)**, and functional **(B)** levels. Only the genera and functions with a relative abundance above 1 and 0.5%, respectively, in at least one sample are shown. Samples included are identified at the bottom of the heatmaps. Colored samples at the bottom correspond to Ob offspring. Colored bars on top of each heatmap represent the delivery mode of the infant with blue corresponding to c-section while orange indicates vaginally delivered.

It has already been established that as an infant develops through the first years of life, so does its gut microbiome, acquiring a greater number of taxa as the child develops. This trend continues until the child’s microbiome reaches an adult-like state at around 2.5 years of age (Clemente et al., 2012; Tamburini et al., 2016). In this study, we observed no significant differences in alpha-diversity (richness and Shannon-Weaver index) between Ob and Nw offspring (data not shown), whereas both measures were correlated with time at both taxonomic and functional levels, as expected (*p* < 0.001). This pattern was clearly observed in the Ob offspring. At the taxonomic level (Figures 2A,C), richness and diversity increased from one week to four months, then decreased at six months, coinciding with the introduction of solid foods, and increased thereafter up to twelve months. At the functional level (Figures 2B,D), both richness and diversity showed an initial sharp increase from one week to two months, and then reached a plateau that slightly decreased from four months to twelve months. This decrease in functional potential in the last few months could be the result of a more functionally homogeneous community amongst samples as the infants got older. Functional alpha-diversity dynamics in Ob offspring (Figures 2B,D) showed a sharper increase from one week to two months, suggesting that samples at one week comprised a single dominant taxon microbiome, like *Bifidobacterium, Escherichia* or *Streptococcus* (Figure 3A) or a set of taxa with similar functional potential.

Richness and diversity trends were slightly different in Nw offspring. At the taxonomic level (Figures 2A,C), both measures increased from one week to two months, decreased slightly up to four months, and then increased up to twelve months. At the functional level (Figures 2B,D), trends in richness paralleled those observed for taxonomic richness. In contrast, functional diversity displayed almost opposite trends, decreasing from one week to four months, increasing at six months, and decreasing again at twelve months. As previously suggested (Valles et al., 2014), these patterns likely imply that the infant’s gut microbiome reaches a high degree of functional complexity early on, with the establishment in the infants’ gut of core taxa such as *Bacteroides, Escherichia* and *Clostridium* and functions such as L-lysine and peptidoglycan biosynthesis.

### Patterns of bacterial dynamics conform to time independently of the mother’s pre-pregnancy status

Although dynamic, the establishment of the GIT microbiota is far from random, as not all GIT-associated organisms have the tools to thrive in the initial stages of gut development (Milani et al., 2017).

To explore whether broad global differential patterns could be discerned between Ob and Nw offspring, we compared taxonomic (only genus level shown) and functional levels across time. Heatmaps showed obvious differences between Ob and Nw offspring at one week that became less apparent at later timepoints (Supplementary Figures S4, S5). The main differences were, on average, a higher relative abundance in Ob offspring of *Bifidobacterium*, *Enterococcus*, and *Streptococcus*, as well as genes related to UMP Biosynthesis I and lysine biosynthesis III pathways; and a higher abundance in Nw offspring of *Klebsiella* and *Escherichia*, and specific pathways such as UDP-N-acetylmuramoyl pentapeptide biosynthesis I (meso-diaminopimelate containing), and peptidoglycan biosynthesis I (meso-diaminopimelate containing) (Figures 3A,B; Supplementary Figures S4, S5).

To assess the level of succession, if any, we examined clustering patterns based on the Bray-Curtis distance. Many studies have suggested the existence of an obesogenic microbiome (Ley et al., 2006; Turnbaugh and Gordon, 2009; Knights et al., 2011; Payne et al., 2011; Bervoets et al., 2013; Le Chatelier et al., 2013; Abenavoli et al., 2019) that might contribute to maternal bacterial translocation during pregnancy (Jimenez et al., 2008; Gosalbes et al., 2013). Considering that maternal obesity significantly increases the probability of early onset obesity in offspring (Harmon and Hannon, 2018), we expected to observe early signs of an obesogenic microbiome throughout the first months of the Ob offsprings’ development, priming the development of a potentially life-spanning obesogenic state. Exploring patterns of clustering at the individual level (using samples from all timepoints for each participant), no specific topology was observed at taxonomic or functional levels in either Ob or Nw offspring, but rather idiosyncratic clustering patterns (data not shown). This is not completely surprising, as earlier microbiome communities are known to be highly dynamic, each exposed to the individual’s own genetic make-up and potentially different environmental factors, resulting in an assemblage of organisms unique to each individual (Milani et al., 2017). Nevertheless, had there been any form of “*in utero*” obesogenic microbial priming, one might have expected some level of clustering along group lines.

Examining clustering patterns at each timepoint revealed that stool samples at one week were mostly dominated by one of several genera (*Escherichia*, *Klebsiella*, *Enterococcus*, *Pantoea*, *Phocaeicola*, *Streptococcus*, *Bifidobacterium*, or *Bacteroides*), and that this was influenced by delivery mode (Figure 3A): all infant’s GIT dominated by *Escherichia* or *Bacteroides* were delivered vaginally, while most infant’s GIT dominated by *Bifidobacterium* were delivered via c-section. The dominant presence of *Bifidobacterium* and *Bacteroides* in the first week of life is not surprising as these genera are known to have the tools to thrive on lactose and HMOs (Marcobal et al., 2010). However, the observation that those dominated by *Bifidobacterium* were mostly delivered via c-section while those dominated by *Escherichia* were vaginally delivered is controversial as most studies agree that *Bifidobacterium* is usually delayed in c-section infants, while *Escherichia* is rather common (Penders et al., 2006; Biasucci et al., 2008).

Comparing Ob versus Nw groups at the one week timepoint, we found that all but one of the infant GIT microbiomes dominated by *Bifidobacterium* were from Ob offspring, whereas all but one of those dominated by *Bacteroides* were from Nw offspring. These results are of interest because they are opposite to previous studies that reported higher abundances of *Bacteroides* and lower of *Bifidobacterium* in Ob offspring (Collado et al., 2010; Patil et al., 2012; Cerdo et al., 2018; Dunton et al., 2020). While the dominance of *Bacteroides* observed in our cohort could be attributed to being vaginally delivered (18 vaginally compared to 4 c-section), the dominance of *Bifidobacterium* is still surprising. Furthermore, our findings could also be revealing differences in microbiome composition due to other factors when compared to other populations such as genetic and geographical differences and/or cultural differences including dietary habits (Patil et al., 2012; Yatsunenko et al., 2012).

With minor topological variations, similar sample clustering patterns by timepoint were observed at the functional and taxonomic levels throughout development, reflecting similarities in the functional potential of taxa present in the community (data not shown). No specific differences were observed at any timepoint between Ob and Nw offspring. As time progressed, single dominance was replaced by shared dominance among several taxa; only *Bifidobacterium* and *Bacteroides* remained dominant overall throughout development. No particular cluster of samples was retained throughout development, suggesting that either colonization of the GIT is not the result of a deterministic succession of specific organisms or functional potential, or that the level of influence on microbiome composition of factors such as maternal microbiome, bacterial load, diet and host genetics, are obscuring successional trends in such a dynamic environment.

**Figure 4:**
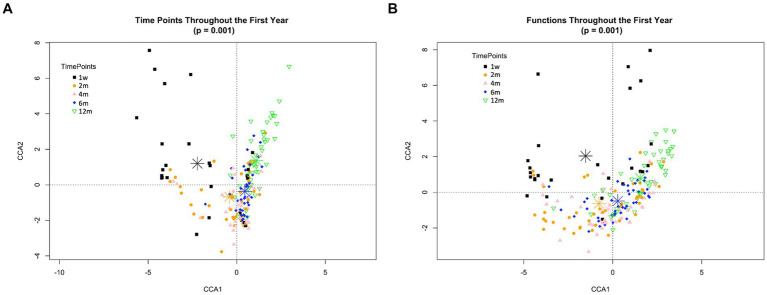
Directed change in taxonomic and functional composition through the infant’s first year of life. Canonical correspondence analysis according to timepoint at both taxonomic **(A)** and functional **(B)** levels. Time points correspond to 1w—one week, 2 m—two months, 4 m—four months, 6 m—six months and 12 m—twelve months. In both cases, one week and twelve months samples are each in a quadrant separated from all other timepoints by both CCA1 and CCA2 axes, while two, four- and six-months samples are clustering closer. Timepoints are differentiated by colors. These patterns were similarly observed both in Nw and Ob offspring independently (data not shown).

Succession was more clearly illustrated at the level of microbiome community structure using Canonical Correspondence Analysis (CCA). This multivariate analysis suggests statistically significant spatial relationships among samples relative to time (*p* < 0.001) but not with respect to the mother’s pre-pregnancy status (*p* = 0.172) (Figure 4). Similar trends were observed at both taxonomic and functional levels (Figures 4A,B). As illustrated by the position of the timepoint centroids, sample assemblages shifted in a unidirectional manner from one week to two, four, six and finally twelve months. The level of heterogeneity between samples also decreased over time (Supplementary Figure S6). This pattern was expected, as the microbial assemblages of infants strongly differ at earlier timepoints, being initially dominated by just one or a few taxa, and progressing towards a more complex, but homogeneous assemblage of many taxa at later timepoints.

### Differential abundance of taxa and functional potential according to pre-pregnancy status

**Figure 5:**
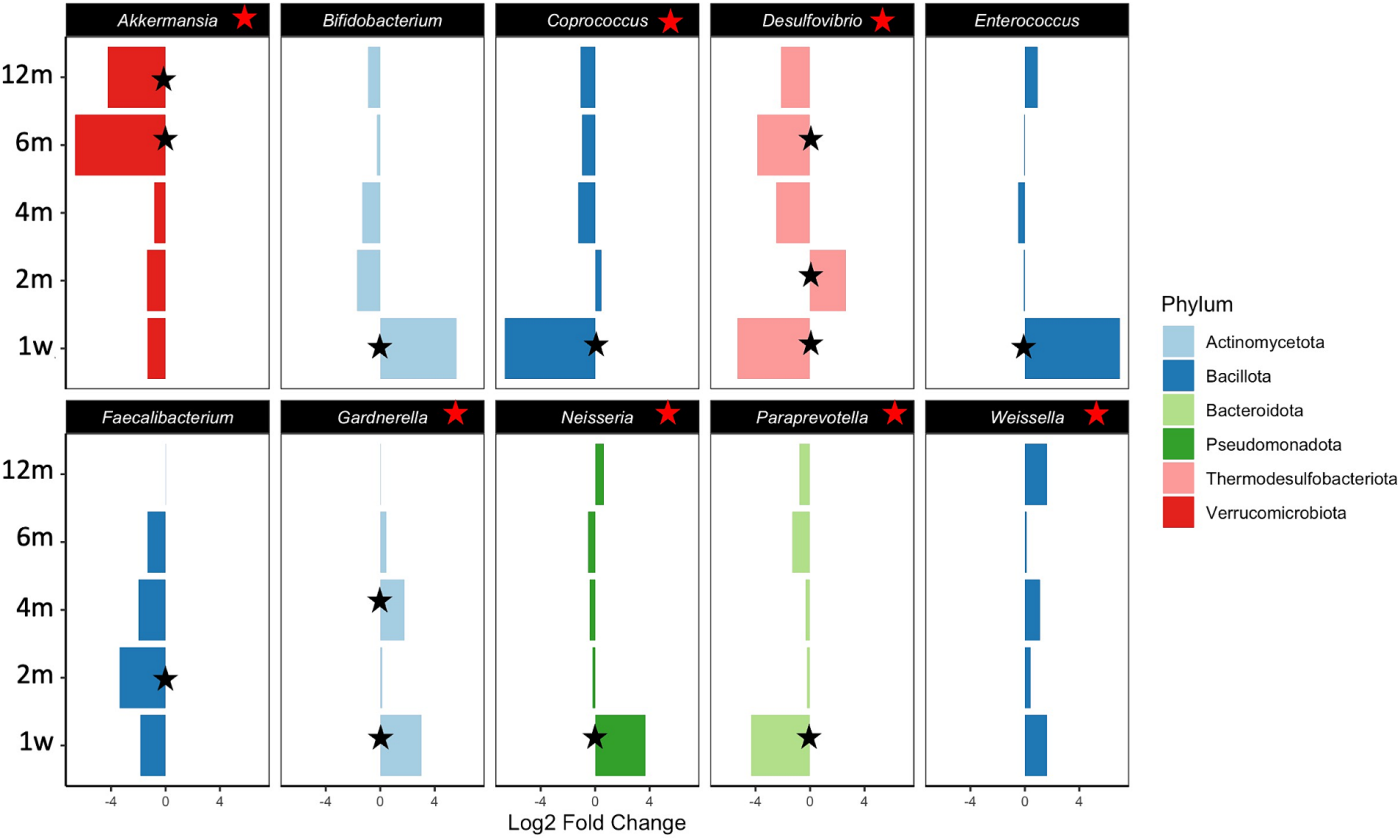
Statistically significant differential abundance of genera between Nw and Ob offspring. Contrasts were executed comparing Ob offspring to Nw offspring. Positive Fold Change reflects enrichment of taxa in Ob versus Nw offspring while negative Fold Change reflects depletion in Ob versus Nw offspring. Red stars indicate significant depletion/enrichment overall (all samples included). Black stars identify specific significance at the particular timepoint indicated. On the y axis abbreviations correspond to timepoints as follows 1w—one week, 2 m—two months, 4 m—four months, 6 m—six months and 12 m—twelve months.

Differential abundances of taxa and functional potential were explored using DESeq2. Contrasts between Ob and Nw offspring, adjusting for timepoint and delivery mode, showed that overall, Ob offspring were significantly depleted in the species *Akkermansia municiphila* and *Desulfovibrio piger* and their respective genera, families, and classes (Table 2; Supplementary Table S4). In contrast, families Clostridiaceae and Xanthomonadaceae, as well as the genera *Weissella*, *Ralstonia*, and *Gardnerella* were enriched (Table 2; Supplementary Table S4; Figure 5). A closer look across timepoints revealed that enriched/depleted fluctuations of the taxa occurred as the infants grew, as seen at the genus level for *Bifidobacterium*, *Clostridium*, *Cronobacter*, *Enterococcus*, *Neisseria* and *Desulfovibrio*. *Akkermansia*, and *Paraprevotella* were consistently depleted in Ob offspring, while *Weissella* and *Gardnerella* were consistently enriched (Table 2; Supplementary Table S4; Figure 5).

**Table 2:** Differential abundances of taxa.

^*^	Overall		One week old		Two months old		Four months old		Six months old		Twelve months old	
	Mean^a^ (FC)^b^		Mean^a^	FC^b^	Mean^a^	FC^b^	Mean^a^	FC^b^	Mean^a^	FC^b^	Mean^a^	FC^b^
**Phylum**												
Verrucomicrobiota	42,576 (−3.08)								42,576.65	−6.79	42,576.65	−4.08
Actinomycetota			7,505,882.46	5.31								
Thermodesulfobacteriota	1,130.95	−1.29	1,130.95	−2.54	1,130.95	1.78						
Bacillota			1,239,722.09	1.65					1,130.95	−2.59		
**Genus**												
*Akkermansia*	14,931.58	−5.96							42,126.50	−6.63		
*Bifidobacterium*			6,495,626.87	5.62								
*Clostridium*			80,737.27	−3.80			80,737.27	2.88	80,737.27	3.39		
*Coprococcus*			4,557.69	−6.65								
*Cronobacter*			4,124.07	3.79								
*Desulfovibrio*	646.84	−2.31	771.66	−5.33								
*Enterococcus*			135,134.74	6.97								
*Faecalibacterium*					114,543.45	−3.39						
*Gardnerella*	69.04	1.53	57.47	3.03			57.47	1.78				
*Lactobacillus*											35,866.66	−4.97
*Neisseria*	1,742.27	−2.50	473.50	3.70								
*Paraprevotella*	656.78	−1.94	739.40	−4.34								
*Prevotella*	112,402.65	−2.04										
*Stenotrophomonas*			1,304.69	4.32								
**Species**												
*Akkermansia muciniphila*	49,692.96	−3.41							49,692.96	−6.42	49,692.96	−3.75
*Bifidobacterium adolescentis*	14,693.74	1.18	14,693.74	2.46								
*Cronobacter sakazakii*	6,183.02	2.88	6,183.02	3.29	6,183.02	3.88	6,183.02	2.62	6,183.02	4.16		
*Desulfovibrio piger*	119.95	−2.54	198.97	−7.70			198.97	−6.67	198.97	−6.05		
*Enterococcus faecalis*			101,973.92	6.87								
*Faecalibacterium prausnitzii*	106,794.00	−1.55			106,794.00	−4.59	106,794.00	−3.32				
*Parabacteroides merdae*	37,439.77	−2.98	37,439.77	−6.79			37,439.77	−5.58				
*Roseburia hominis*	5,631.72	−1.45	5,631.72	−3.62							5,631.72	−2.27
*Ruminococcus gnavus*					133,581.38	3.52			133,581.38	3.46		
*Veillonella atypica*	19,763.91	0.93	19,763.91	3.75							19,763.91	1.96

Selected taxa identified as significantly depleted or enriched in one or more timepoints with an adjusted *p* value < 0.05. Fold Change reflects contrasts of Ob versus Nw offspring, with positive values reflecting enrichment of taxa in Ob compared to Nw offspring and negative values a depletion in Ob compared to Nw offspring.

* Taxa selected amongst those that had a significantly differential abundance with a *p*-adjusted value < 0.05 for the differential analysis.

a Mean values refer to mean normalized counts of taxa according to the pre-pregnancy status of the mother.

b FC refers to the log2 fold change per unit of change.

Although these results largely validate findings from other studies, the significant enrichment of *Bifidobacterium* at one week and the overall depletion of the genus *Desulfovibrio* and its species in Ob offspring compared to Nw offspring are particularly intriguing. These patterns contrast with previous reports, where *Bifidobacterium* is negatively correlated, while *Desulfovibrio* and its species are positively correlated with obesity (Gao et al., 2015; Bai et al., 2019; Da Silva et al., 2020; Palmas et al., 2021; Singh et al., 2023).

Perhaps the most relevant result is the statistically significant depletion of the genus *Akkermansia* and its species *Akkermansia municiphila* in Ob offspring. The statistically significance of the findings is underscored by two main points: first, *Akkermansia* has been identified as a biomarker that is negatively associated with obesity, a fact that is consistent in the literature (Derrien et al., 2004; Rodrigues et al., 2022), and second, our study identified no other broad pattern suggestive of an innate and life-spanning difference between Ob and Nw offspring. Consistent with previous reports that *Akkermansia* is a late colonizer of the gut (Valles et al., 2014; Suarez-Martinez et al., 2023), temporal analysis of our data revealed that *Akkermansia* was not significantly depleted relative to Nw offspring until they were six and twelve months old, implying that its establishment in the gut is delayed in Ob offspring. This suggests that mechanisms may already be in place early on that hinder the colonization of this bacterium in the GIT of Ob offspring. Furthermore, the species *Faecalibacterium prausnitzii*, also considered as a biomarker of gastrointestinal health (Parsaei et al., 2021), was depleted in Ob compared to Nw offspring. Whether these differences result from the lack of the appropriate succession of gut microbiota that creates the suitable environment for settlement of these key species, or the influence of other maternal or environmental/epigenetic factors, is yet to be determined.

At the functional level, statistically significant differences in pathway abundances were observed at the timepoint level, particularly at one week, where 40 pathways showed statistically significantly differential abundance (23 depleted and 17 enriched) in Ob offspring compared to Nw offspring. At two months eight pathways were differentially abundant (two depleted and six enriched), while at four and six months, one and two pathways were statistically significantly depleted, respectively, (selected pathways shown in Table 3).

**Table 3:** Differential abundances of functions/pathways.

Pathway	Mean^a^	Log2FC	lfcSE	*p*-value	*p*-adj	Tp^*^
PWY-7312: dTDP-β-D-fucofuranose biosynthesis	10.12	−11.50	2.41	<0.001	<0.001	Overall
UDPNACETYLGALSYN-PWY: UDP-N-acetyl-D-glucosamine biosynthesis II	13.95	23.46	3.23	<0.001	<0.001	1w
PWY-7294: D-xylose degradation IV	24.26	22.14	3.81	<0.001	<0.001	1w
PWY-7245: superpathway of NAD/NADP - NADH/NADPH interconversion (yeast)	60.09	−19.67	2.66	<0.001	<0.001	1w
PWY0-881: superpathway of fatty acid biosynthesis I (*E. coli*)	10.80	30.00	8.14	<0.001	<0.001	1w
PWY-6387: UDP-N-acetylmuramoyl pentapeptide biosynthesis I (meso-diaminopimelate containing)	5,228.33	0.37	0.11	<0.001	0.02	1w
PEPTIDOGLYCANSYN-PWY: peptidoglycan biosynthesis I (meso-diaminopimelate containing)	5,159.31	0.36	0.12	<0.001	0.03	1w
PWY-6386: UDP-N-acetylmuramoyl-pentapeptide biosynthesis II (lysine-containing)	5,288.30	0.39	0.12	<0.001	0.02	1w
PWY-2942: L-lysine biosynthesis III	5,435.34	0.44	0.15	<0.001	0.03	1w
PWY-5156: superpathway of fatty acid biosynthesis II (plant)	21.47	−49.82	3.04	<0.001	<0.001	2 m
PWY-7294: D-xylose degradation IV	24.26	30.79	3.00	<0.001	<0.001	2 m
PWY-8173: anteiso-branched-chain fatty acid biosynthesis	12.74	48.13	5.08	<0.001	<0.001	2 m
PWY-8174: odd iso-branched-chain fatty acid biosynthesis	12.74	48.13	5.08	<0.001	<0.001	2 m
PWY-8175: even iso-branched-chain fatty acid biosynthesis	12.74	48.13	5.08	<0.001	<0.001	2 m
PWY-6807: xyloglucan degradation II (exoglucanase)	58.21	25.43	5.55	<0.001	<0.001	2 m
LIPA-CORESYN-PWY: lipid A-core biosynthesis (*E. coli* K-12)	68.85	−5.88	1.59	<0.001	0.01	2 m
P125-PWY: superpathway of (R,R)-butanediol biosynthesis	84.53	3.32	0.95	<0.001	0.03	2 m
PWY-7200: superpathway of pyrimidine deoxyribonucleoside salvage	37.09	−21.97	3.86	<0.001	<0.001	4 m
PWY-7200: superpathway of pyrimidine deoxyribonucleoside salvage	37.09	−21.86	3.78	<0.001	<0.001	6 m
PWY0-881: superpathway of fatty acid biosynthesis I (*E. coli*)	10.80	−26.50	6.31	<0.001	0.01	6 m

Pathways selected amongst those that had a significantly differential abundance with a *p*-adjusted value < 0.05 for the pathway differential analysis. Positive Fold Change reflects enrichment of pathways in Ob compared to Nw offspring while negative Fold Change reflects depletion in Ob compared to Nw offspring.

* Tp refers to the timepoint at which the DESeq analysis was run as follows 1w = one week, 2 m = two months, 4 m = four months, 6 m = six months, 12 m = twelve months.

a Mean values refer to mean normalized counts of pathway counts according to the pre-pregnancy status of the mother.

**Figure 6:**
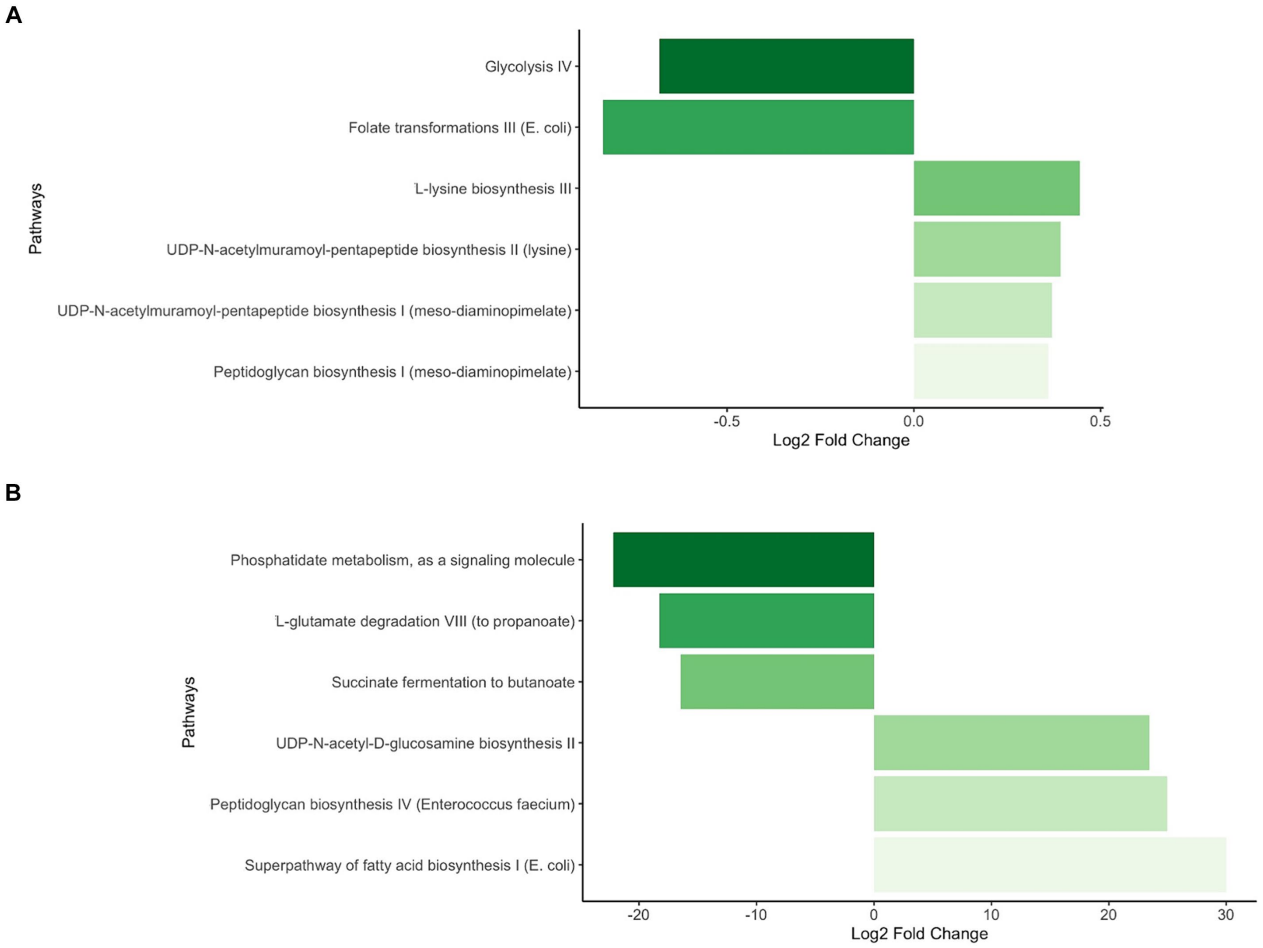
Statistically significant differential abundance of pathways between Ob and Nw offspring at one week of age. Contrasts were executed comparing Ob offspring to Nw offspring. Positive Fold Change reflects enrichment of pathways in Ob compared to Nw offspring while negative Fold Change reflects depletion in Ob compared to Nw offspring All pathways depicted had a *p*-adjusted <0.05. **(A)** Selected pathways with minor but significant differential abundance. **(B)** Selected pathways with major significant differential abundance.

Interestingly, at one week functional differentials revealed that Ob offspring were enriched, amongst a few others, in the UDP-N-acetyl-D-glucosamine biosynthesis II pathway (Figure 6; Table 3). This pathway affects the production of leptin (Wang et al., 1998), an adipocyte hormone (also called OB protein) that signals the brain, resulting in lower food intake and adipose development. Yet, studies in mice and humans have shown that blood levels of leptin are higher in overweight/obese individuals, suggesting that these individuals have developed a leptin resistant disorder (Maffei et al., 1995; Wang et al., 1998). Studies have also demonstrated that maternally produced leptin plays an important role during pregnancy and in fetal growth and development, and that maternal obesity is correlated to higher maternal leptin serum levels in blood, increased concentration in the umbilical cord and placenta. Surprisingly, the vast majority of the maternally produced leptin in placenta is not released into fetal circulation (Linnemann et al., 2000). The latter raises two fundamental questions, do maternal leptin serum levels act as signaling molecules influencing the fetal’s microbiome and are infants exposed to higher levels of maternal and/or fetal leptin more likely to develop leptin resistance at some stage in their early life? As leptin regulates appetite and energy expenditure, leptin resistance during infancy could set the stage for the development of early onset metabolic diseases, such as obesity.

Also noteworthy at this timepoint is the enrichment in the UDP-N-acetylmuramoyl pentapeptide biosynthesis II (lysine containing) and UDP-N-acetylmuramoyl pentapeptide biosynthesis I (meso-diaminopimelate containing) pathways, as they play central roles in the maturation of bacterial membrane peptidoglycans (PGN). PGNs have microbe-associated molecular patterns that are recognized and activated in the host’s pattern recognition receptors (PRR) such as the NOD-like receptors NOD1 and NOD2. These contribute to the homeostatic activation and regulation of the metabolic and immune systems (Fernandez-Garcia et al., 2021). Interactions between the bacterial PGN and the NOD-like PRR are of great importance because in the early stages of infancy, this crosstalk will enable adequate maturation of the immune system to accurately differentiate “friends from foes” (Chamaillard et al., 2003). However, overexpression of NOD1 has also been associated with inflammation, as well as metabolic diseases such as diabetes and obesity (Zangara et al., 2021). It would be interesting to determine whether the differential abundance of both UDP-N-acetylmuramoyl pentapeptide biosynthesis pathways could be correlated with an overexpression of NOD1 in the Ob offspring, leading to an early inception of inflammation.

## Is there an obesity microbial primming set in from the start?

Our study investigated the GIT microbiome of 46 Emirati infants during their first year of life. This study is the first of its kind in the UAE and the GCC region, filling a significant gap in this field of research. Although our study provided initial insights into the developing microbiome of infants in two different health contexts, a limiting factor was the lack of access to maternal stool samples. Access to the latter would have enabled us to explore potential correlations between GIT microbial composition of mothers and their offspring, and to detect possible microbial translocation events from mother to infant. To further investigate potential obesogenic microbiome-related factors that may be established early in life, particular focus should be placed on early timepoints. This is crucial because later, confounding effects from the environment factors, diet, antibiotic consumption, or other influences, might conceal initial patterns of microbial transmission.

Our study nevertheless revealed interesting results when exploring the possibility that the mother’s pre-pregnancy status might influence the development and establishment of her offspring’s GIT microbiome. We found that above all, throughout the first year of life, the infants’ GIT microbial community is first and foremost influenced by time and delivery mode. Obesity markers, on the other hand, are revealed at deeper levels, in particular, the significantly higher Bacillota/Bacteroidota ratio observed in the Ob offspring GIT microbiome, the significant depletion in the genus *Akkermansia* and the species *Akkermansia municiphila* and *Faecalibacterium prausnitzii*, and the enrichment on the UDP-N-acetyl-D-glucosamine biosynthesis II and the UDP-N-acetylmuramoyl pentapeptide biosynthesis pathways. More specifically, the potential increase of leptin production in Ob offspring as a result of the UDP-N-acetyl-D-glucosamine biosynthesis II pathway enrichment and whether the maternal’s leptin could serve as a “paracrine” signaling molecule to the fetus’s GIT microbiome should be further explored, as increased leptin serum levels could be determinant in the early development of the leptin resistance disorder. Furthermore, these findings are significant, as despite the lack of microbial deterministic patterns of succession in Ob and Nw offspring, some underlaying mechanism(s) must be in place early on, determining punctual but key differences between Ob and Nw offspring’s GIT microbiomes. Whether these are due to the mother’s contribution through her potentially obesogenic microbiome, hormone levels, or other epigenetic effects needs further exploration.

## Data availability statement

The data presented in this study is available in the SRA repository accession number PRJNA1130109.

## Ethics statement

This study involving human subjects was approved by the Dubai Scientific Research Ethics Committee, Dubai Health Authority and the NYUAD Institutional Review Board, New York University Abu Dhabi. The study was conducted in accordance with the local legislation and institutional requirements. Written informed consent was obtained from all participants. Written consentt was also obtained from the minor(s)’ legal guardian/next of kin, for collecting their stool samples.

## Author contributions

YV: Conceptualization, Data curation, Formal analysis, Funding acquisition, Investigation, Methodology, Project administration, Resources, Supervision, Validation, Visualization, Writing – original draft, Writing – review & editing. MuA: Data curation, Formal analysis, Methodology, Software, Writing – original draft, Writing – review & editing. MaA: Investigation, Methodology, Writing – review & editing. CI: Conceptualization, Funding acquisition, Writing – review & editing. AmA: Methodology, Formal analysis, Writing – review & editing. ND: Data curation, Formal analysis, Methodology, Software, Writing – review & editing. KG: Writing – review & editing. RA: Writing – review & editing. MT: Investigation, Resources, Writing – review & editing. AbA: Conceptualization, Funding acquisition, Investigation, Project administration, Supervision, Validation, Writing – review & editing.
