# Supplementary material for: The infants’ gut microbiome: setting the stage for the early onset of obesity
Source: Front Microbiol. 2024 Jul 16;15:1371292. doi: 10.3389/fmicb.2024.1371292 (PMC11287775; doi:10.3389/fmicb.2024.1371292)
Supplement: Supplementary file 1 [file Data_Sheet_1.PDF]

Supplementary Material

Supplementary Figures and Tables

Supplementary Table 1. Breakdown of samples included in the study.

	[1]Nw offspring	[2]Ob offspring	Total
Total Number of Participants (vaginal/C-section)	23(18/4/1NA)	23(9/14)	46
Number at one week old	11	16	27
Number at two months old	22	21	43
Number at four months old	21	21	42
Number at six months old	22	23	45
Number at twelve months old	23	23	46

[1] Normoweight

[2] Obese

Supplementary Table 2. Summary of number of reads before and after QC in millions of sequences

Sample size: 203

	Pre QC (Million seqs)	Post QC (Million seqs)
Min.	5.7	0.500
1st Qu.	8.20	7.900
Median	9.00	8.700
Mean	9.08	8.533
3rd Qu.	9.70	9.550
Max.	18.80	15.500

Supplementary Table 3. Estimated effect with corresponding 95% CI. Statistically significant effects (95% CI) are shown in bold.

Phyla	Pre-pregnancy status	Gender	Delivery mode
Actinomycetota	-0.76 (-16.189, 8.158)	-0.009 (-15.562, 8.282)	-7.271 (-16.371, 1.829)
Bacillota	0.196 (-13.2, 5.933)	-0.719 (-12.795, 4.615)	**-7.462 (-13.316, -1.609)**
Bacteroidota	0.896 (4.958, 9.545)	2.84 (5.564, 10.884)	**13.61 (4.779, 22.44)**
Pseudomonadota	0.44 (-6.866, 8.763)	-2.191 (-6.278, 5.544)	1.457 (-7.033, 9.947)
Fusobacteriota	0.003 (-0.007, 0.018)	-0.001 (-0.006, 0.013)	0.008 (-0.008, 0.024)
Verrucomicrobiota	**-1.054 (-1.438, -0.044)**	0.15 (-1.368, 1.089)	-0.429 (-1.460, 0.603)

Supplementary Table 4. **Differential abundances of taxa.** Selected taxa identified as significantly depleted or enriched in one or more timepoints with an adjusted p value < 0.05. Fold Change reflects contrasts of Ob offspring versus Nw offspring, with positive values reflecting enrichment of taxa in Ob versus Nw offspring and negative values a depletion in Ob versus Nw offspring.

*	Overall		1-week-old		Two months old		Four months old		Six months old		Twelve months old	
	[1]Mean	[2]FC	[1]Mean	[2]FC	[1]Mean	[2]FC	[1]Mean	[2]FC	[1]Mean	[2]FC	[1]Mean	[2]FC
Phylum												
Verrucomicrobiota	42576.65	-3.08							42576.65	-6.79	42576.65	-4.08
Actinomycetota			7505882.46	5.31								
Thermodesulfobacteriota	1130.95	-1.29	1130.95	-2.54	1130.95	1.78						
Bacillota			1239722.09	1.65					1130.95	-2.59		
Class												
Actinomycetes			7819926.56	5.11								
Betaproteobacteria					37863.02	3.35						
Desulfovibrionia	562.08	-2.07	473.34	-3.18					473.34	-2.66	473.34	-1.73
Negativicutes			284803.51	2.85								
Verrucomicrobiae	39981.24	-3.26							39981.24	-6.91	39981.24	-4.18
Order												
Alteromonadales							682.76	1.57				
Bifidobacteriales			6823558.63	5.31								
Desulfovibrionales	613.00	-1.76	719.63	-3.59					719.63	-3.01		
Neisseriales			982.35	3.48								
Veillonellales			282050.52	3.87								
Verrucomicrobiales	42172.74	-3.25							42172.74	-6.81		
Family												
Akkermansiaceae	40024.76	-3.19							40024.76	-6.7		
Bacillaceae			4045.58	2.26								
Bifidobacteriaceae			6434242.97	5.22								
Clostridiaceae									116377.08	3.5		
Coprobacillaceae			53367.75	-5.17								
Desulfovibrionaceae	618.84	-1.92	618.84	-3.46					618.84	-2.78		
Enterococcaceae			130730.82	6.5127								
Neisseriaceae			901.84	4.49								
Sutterellaceae			13375.37	-5.61	13375.37	5.46						
Veillonellaceae			293284.24	4.38								
Genus												
Akkermansia	14931.58	-5.96							42126.50	-6.63		
Bifidobacterium			6495626.87	5.62								
Clostridium			80737.27	-3.80			80737.27	2.88	80737.27	3.39		
Coprococcus			4557.69	-6.65								
Cronobacter			4124.07	3.79								
Desulfovibrio	646.84	-2.31	771.66	-5.33								
Enterococcus			135134.74	6.97								
Faecalibacterium					114543.45	-3.39						

Gardnerella	69.04 1.53	57.47 3.03		57.47 1.78		
Lactobacillus						35866.66 -4.97
Neisseria	1742.27 -2.50	473.50 3.70				
Paraprevotella	656.78 -1.94	739.40 -4.34				
Prevotella	112402.65 -2.04					
Stenotrophomonas		1304.69 4.32				
Species						
Akkermansia muciniphila	49692.96 -3.41				49692.96 -6.42	49692.96 -3.75
Bifidobacterium adolescentis	14693.74 1.18	14693.74 2.46				
Cronobacter sakazakii	6183.02 2.88	6183.02 3.29	6183.02 3.88	6183.02 2.62	6183.02 4.16	
Desulfovibrio piger	119.95 -2.54	198.97 -7.70		198.97 -6.67	198.97 -6.05	
Enterococcus faecalis		101973.92 6.87				
Faecalibacterium prausnitzii	106794.00 -1.55		106794.00 -4.59	106794.00 -3.32		
Parabacteroides merdae	37439.77 -2.98	37439.77 -6.79		37439.77 -5.58		
Roseburia hominis	5631.72 -1.45	5631.72 -3.62				5631.72 -2.27
Ruminococcus gnavus			133581.38 3.52		133581.38 3.46	
Veillonella atypica	19763.91 0.93	19763.91 3.75				19763.91 1.96

*Taxa selected amongst those that had a significantly differential abundance with a **p-adjusted value < 0.05** for the differential analysis.

[1]**Mean** values refer to mean normalized counts of taxa according to the pre-pregnancy status of the mother.

[2]**FC** refers to the log2 fold change per unit of change.

Supplementary FIGURE 1

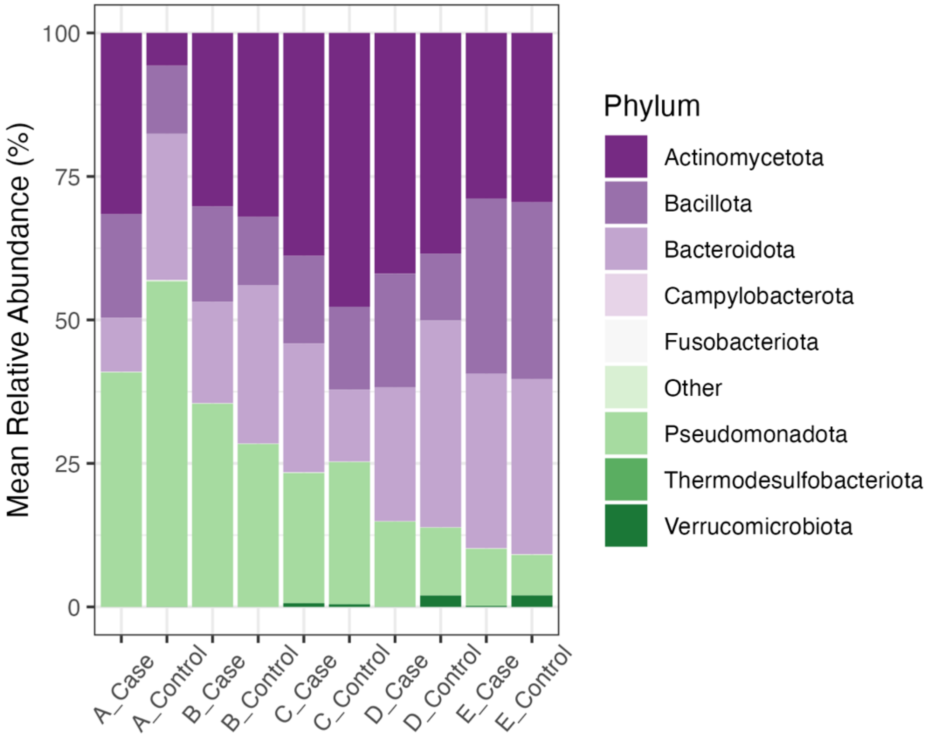

Supplementary Figure 1. Barplot depicting the relative abundance of the phyla per timepoint and type of sample (pre-pregnancy status, Nw offspring (Case) versus Ob offspring (Control)). Only the most abundant phyla are shown with the remainder being clustered as other. Denominations on the x-axis correspond to the following timepoints A = 1 week, B = 2 months, C = 4 months, D = 6 months, E = 12 months.

Supplementary FIGURE 2

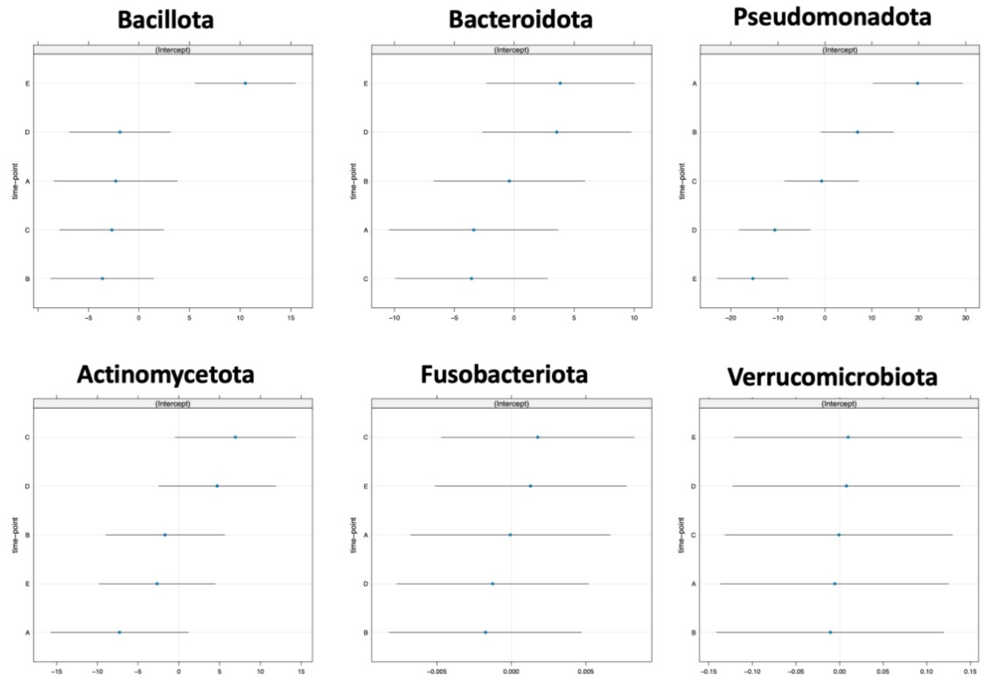

Supplementary Figure 2. This figure shows the estimated intercept with 95% CI for each phyla by timepoint. The twelve months (E) timepoint was statistically significantly higher than the average for Bacillota. However, for Pseudomandata, 6 months and 12 months timepoints were significantly lower than the average while the one-week timepoint was statistically significantly higher than the average. No statistically significant differences were observed for the remainder phyla. Denominations on the y-axis correspond to the following timepoints A = 1 week, B = 2 months, C = 4 months, D = 6 months, E = 12 months.

Supplementary FIGURE 3

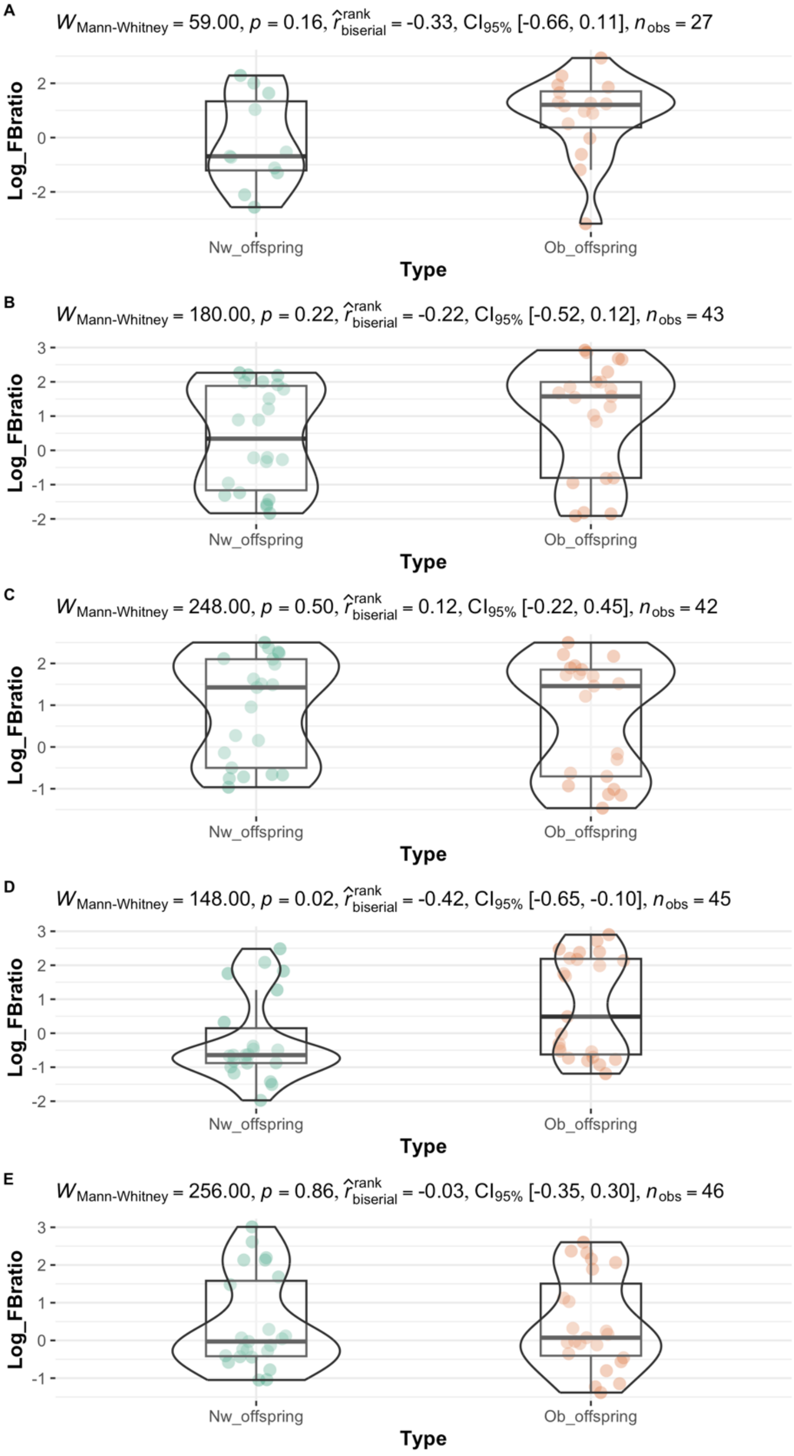

Supplementary Figure 3: Bacillota/Bacteroidota ratio comparisons between Ob and Nw offspring at each timepoint. (A) 1 week; (B) 2 months, (C) 4 months, (D) 6 months, (E) 12 months.

Supplementary FIGURE 4

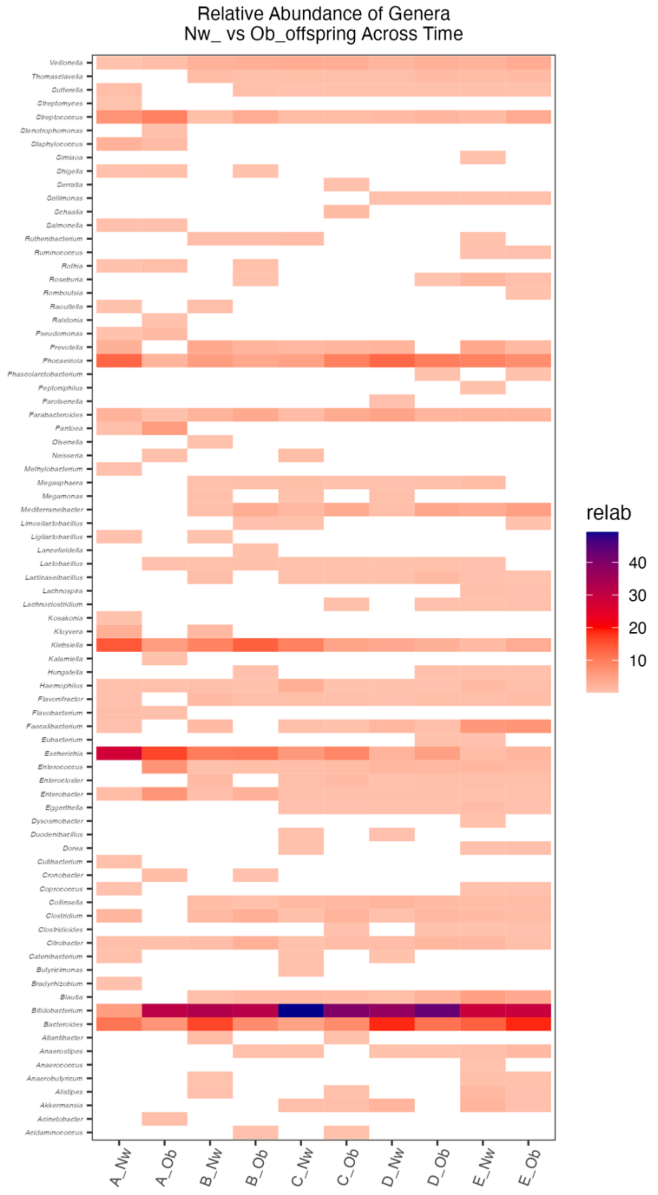

Supplementary Figure 4. Heatmap depicting composition per timepoint and type of sample (pre-pregnancy status, Nw offspring vs. Ob offspring), at taxonomic level. Only the genera with a relative abundance above 1% in at least one sample are shown. Timepoint and type of samples included in each group are identified at the bottom of the heatmap. Denominations on the x-axis correspond to the following timepoints A = 1 week, B = 2 months, C = 4 months, D = 6 months, E = 12 months.

Supplementary FIGURE 5

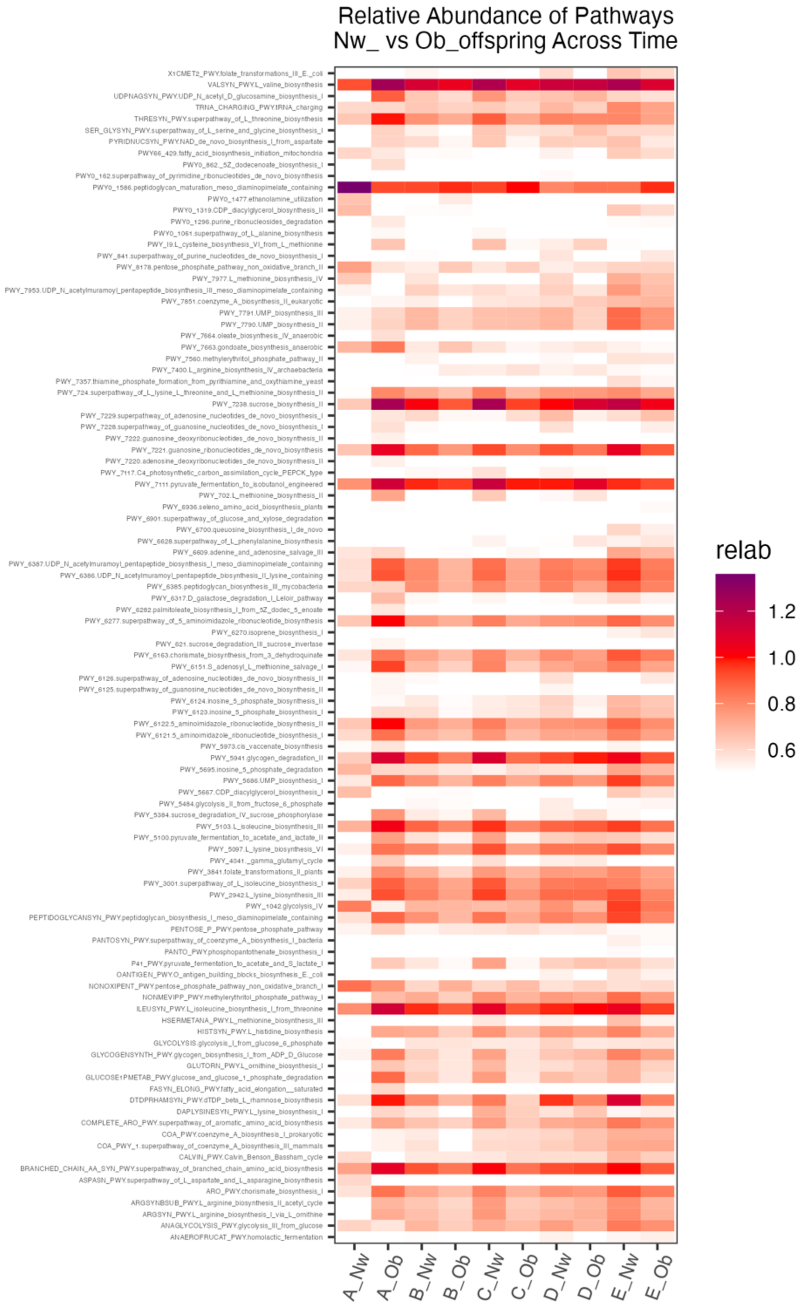

Supplementary Figure 5. Heatmap depicting composition per timepoint and type of sample (pre-pregnancy status, Nw offspring vs. Ob offspring), at the functional level. Only the functions with a relative abundance above 0.5% in at least one sample are shown. Timepoint and type of samples included in each group are identified at the bottom of the heatmap. Denominations on the x-axis correspond to the following timepoints A = 1 week, B = 2 months, C = 4 months, D = 6 months, E = 12 months.

Supplementary FIGURE 6

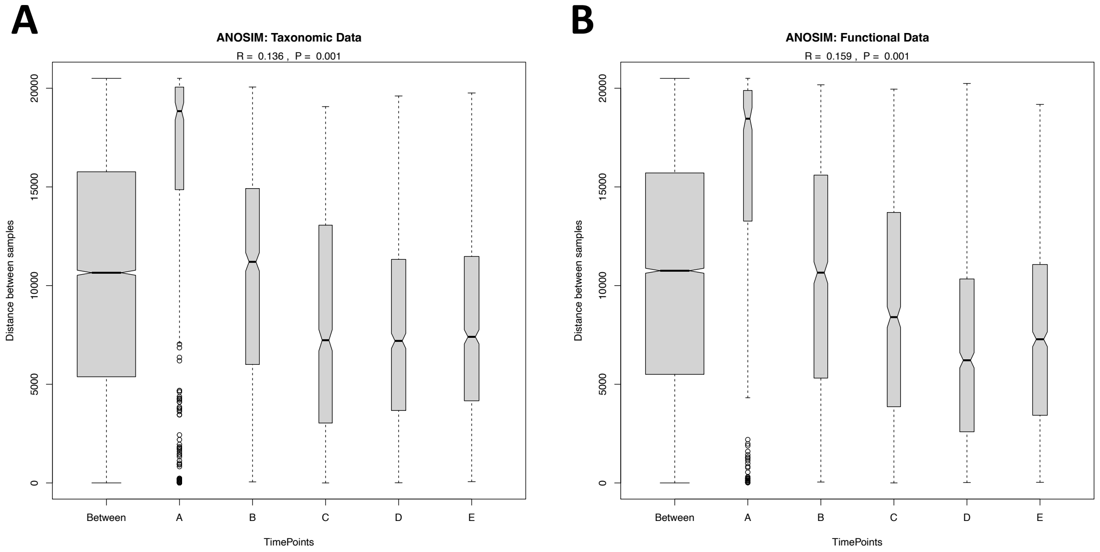

Supplementary Figure 6. Comparison of timepoints at the taxonomic (A) and functional (B) levels through implementation of an ANOSIM analysis. The length of boxplots indicates the level of heterogeneity of the samples within each timepoint while its width indicates the number of samples compared for each timepoint. As time goes by, samples within each timepoint become less heterogeneous. Denominations on the x-axis correspond to the following timepoints A = 1 week, B = 2 months, C = 4 months, D = 6 months, E = 12 months.
